# Navigating dual pressures: The impact of environmental policies and market demand risks on the sustainable development of green building materials - A case study of the green cement industry

**DOI:** 10.1016/j.heliyon.2025.e41942

**Published:** 2025-01-16

**Authors:** Wenxuan Guo

**Affiliations:** Department of Earth Science and Engineering, Taiyuan University of Technology, Taiyuan, Shanxi Province, 030000, China

**Keywords:** Sustainable development, Green building materials, Environmental policies, Market demand risks, Multi-stage game, Genetic algorithm, Backward induction method

## Abstract

This paper addresses the issue of sustainable development in the green building material industry under the dual pressures of environmental policies and market demand risks. A multi-stage game model is developed to explore the strategic interactions between green material producers and intermediate suppliers in decision-making related to green research and product pricing. Utilizing backward induction methods, genetic algorithm simulations, and production data from the cement industry, we analyze the sensitivity of product greenness, pricing, sales volume, and profits to changes in market demand risks under both strict and lenient environmental policies. The findings indicate that strict environmental policies are more effective than lenient ones in promoting the sustainable development of the green building materials sector. Strict policies encourage companies to engage in green research and production while helping to maintain sales and prices, thereby ensuring profitability—especially when competing products have lower levels of greenness. This paper adds a new perspective by considering the dual pressures of environmental policies and market demand risks and their interactions, aiming to provide insights for related policymaking.

## Highlights

• Developed a multi-stage game model to explore sustainable strategies in green building materials industry.
• Analyzed interactions between green material producers and intermediate suppliers in R&D and pricing.
• Used backward induction and genetic algorithms to assess product greenness, pricing, sales, and profits.
• Found strict environmental policies foster green R&D and ensure industry sustainability.
• Strict policies help maintain product sales and profitability, especially with less eco-friendly competition.

## 1 Introduction

Green building materials are products that consume fewer resources and energy throughout their lifecycle, have minimal ecological impact, and exhibit characteristics such as energy efficiency, reduced emissions, low carbon footprint, safety, convenience, and recyclability. Currently, the building material industry is a substantial contributor to carbon emissions. For instance, the cement production process alone accounts for approximately 7 % of global carbon emissions [1]. If considered as a separate entity, the global cement industry is the third largest carbon emitter after China and the United States. China produces nearly 60 % of the world's cement and consistently holds the top position in output [2]. However, the quality and branding of Chinese cement do not fully align with its production volume, and the sector's carbon emissions constitute around 15 % of the nation's total emissions, exceeding half of the global cement industry's emissions [1,3]. In response, the Chinese government has released the “Implementation Plan for High-Quality Development of the Green Building Material Industry” [4] indicating that the development of green building materials is a key direction for the industry's transformation and a necessary choice for supply-side structural reform.

The sustainable development of the green building material industry requires that the improvement in the greenness of building materials does not undermine their sales and corporate profits. In recent years, the increasing market demand risks have posed new challenges for operating green building material companies and formulating effective environmental policies. With the changing global economic situation, the Chinese real estate market has cooled, and the uncertainty in the demand for building materials has also increased. The shrinking market demand has put considerable survival pressure on building material manufacturers and created substantial resistance to their green transformation. For example, according to data disclosed by the China Cement Industry website [5], as market demand has contracted, the average price of cement over the past three years has dropped from approximately 650 yuan per ton to a minimum of 320 yuan per ton; the price of cement clinker has followed a similar downward trend. To continue advancing the green transformation of the building material industry, the Chinese government launched a pilot project in 2020 to integrate low-carbon indicators into government procurement standards for building materials and promote the use of green building materials to improve construction quality. The first batch of pilot cities included six, and by 2022, this number expanded to 48, with the target of reaching no fewer than 100 pilot cities by 2026. The government is the main single consumer of building materials, with approximately one-third of cement being purchased by the government. By integrating low-carbon indicators into government procurement policies for green building materials in pilot areas, the government can effectively absorb the green premium and send a signal to the market for green-cement demand. At the same time, the pilot policies impose stricter requirements on the greenness of building materials, increasing competition and pressure on related manufacturers to upgrade their products’ environmental performance [6], which can be viewed as “strict environmental policies.” In non-pilot areas, government procurement standards for building materials are relatively lenient, and companies face less pressure in terms of green investment, which can be considered “lenient environmental policies.” Empirical studies have pointed out that the effects of environmental regulations on green innovation are contextualized and diverse, including enhancing, stagnating, undermining, U-shaped, and inverted U-shaped patterns [7]. Whether strict environmental policies in pilot areas can effectively promote energy-saving and emission-reduction efforts by building material manufacturers, and whether these policies will adversely affect business operations given the current high risks in market demand, remain open questions. Which policy is more beneficial for helping building material companies cope with future market demand risks and promoting the sustainable development of the green building material industry and enterprises? These issues need to be urgently explored.

**Fig. 1:**
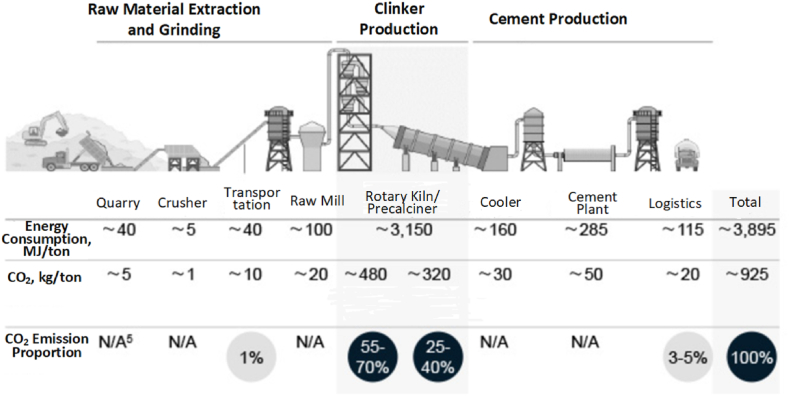
Carbon dioxide emissions at each stage of cement production.

The operation of the green building materials sector involves interactive decision-making among companies in the supply chain. Environmental policies and market demand risks directly affect these interactions, further influencing the sustainability of both businesses and the industry [8]. For instance, in the cement industry, production includes three main stages: raw material extraction, clinker production, and cement manufacturing, as shown in Fig. 1. Data from the Global Cement and Concrete Association reveal that clinker production accounts for about 95 % of carbon dioxide emissions [9]. Current efforts to develop low-carbon cement focus on modifying the mineral composition of traditional silicate cement clinker. Researchers are working on creating low-calcium or calcium-free clinker systems, which can lower carbon dioxide emissions by reducing the use of calcium carbonate [10]. Cement clinker production lines are generally established in regions rich in limestone resources. For example, Conch Cement, which ranks second in clinker production capacity in China, mainly builds its cement clinker production lines in areas along the Yangtze River, where limestone resources are abundant. With the price advantage of water transport, clinker is sold to regions with high cement demand but limited limestone resources. The cement demand market is fragmented, and to reduce transportation costs, the main downstream cement production entities are cement grinding stations scattered across various locations. Clinker production is a key link in energy-saving and emission-reduction efforts [11]. The energy-saving and emission-reduction investments made by clinker production companies directly determine the greenness of intermediate products (cement clinker), which in turn affects the greenness of downstream cement products and their market competitiveness under environmental policies. Downstream cement producers determine the final cement price based on clinker procurement costs, greenness, and market demand and competition conditions and sell it to construction companies. Cement sales directly impact the profits of producers and suppliers, thereby affecting both parties' pricing decisions and the suppliers' green research and development decisions. Market demand risks directly influence the potential market demand for cement, which in turn affects its sales volume. Different environmental policies influence the sales of specific cement products by affecting the market's sensitivity to the greenness and price of cement products [12,13]. Specifically, strict environmental policies in pilot areas increase market sensitivity to the greenness of building materials, while under lenient environmental policies, the market tends to be more sensitive to cement prices. In summary, environmental policies and market demand risks influence the interactive decisions between green building material suppliers and producers on green R&D and product pricing, thereby affecting the sustainable development of related enterprises and the industry. Therefore, modeling and analyzing the impacts of environmental policies and market demand on the sustainable development of the green building material industry within the context of interactive decision-making is necessary.

(Source: Cement Industry Energy and Carbon Dioxide Performance: Getting the Numbers Right)

The existing literature on green building materials highlights the complexities of supply chain interactions, often examined through game theory. Researchers such as Zhang and Du [14] have developed comprehensive performance evaluation systems for the green building supply chain by effectively integrating objective entropy methods with subjective analytic hierarchy processes. This research is essential as it establishes a foundational framework for understanding the possible interactions and optimizations within the sector. Furthermore, game theory and simulation analyses have been employed to explore how market risks influence decision-making in the green supply chain. Agi et al. [15] conducted a systematic review of game theory-based models, emphasizing the effects of market dynamics on the strategic choices made by supply chain participants. Their findings are crucial, illustrating how external market uncertainties can affect the adoption of green practices and materials. Additionally, researchers such as Liu et al. [16] have examined the impact of various environmental policies within game-theoretical frameworks. They have focused on government regulations designed to coordinate emission reductions among enterprises in the green supply chain, revealing that well-structured incentives and regulatory frameworks can significantly shape corporate sustainability strategies. Gao et al. [17] utilized evolutionary game theory to simulate how dynamic government subsidies influence green building development, showing that phasing out subsidies could foster autonomous growth in green buildings, provided effective government oversight and punitive measures are in place. Collectively, these studies have emphasized the vital role of game theory and simulation analyses in understanding the intricate decision-making processes within the green building materials supply chain. However, they have often addressed only single scenarios concerning market risks or environmental policies, overlooking the complex challenges arising from the dual pressures of environmental policies and market demand risks on the dynamics of the green building materials supply chain.

This study investigates the interaction decision-making processes of green building material producers and suppliers under the dual pressures of environmental policies and market demand risks. The aim is to determine whether strict or lenient environmental policies better assist companies in addressing market demand risks and promote the green building sector's sustainable development. The theoretical and practical contributions of this research are twofold. Theoretically, it offers a new perspective for analyzing the combined effects of environmental policies and market demand risks on supply chain decisions. It reveals the interplay between environmental policies and market risks as well as their impacts on supply chain stability, thereby enriching the theoretical foundation of green building supply chain management. Practically, the study provides specific policy recommendations to help governments develop more effective environmental policies that support the sustainable development of the green building industry, particularly in contexts of high market uncertainty. Additionally, it offers decision-making support for managers in the building materials sector, enabling them to adjust their business strategies according to varying environmental policies and market conditions, ultimately enhancing their market adaptability and competitiveness.

The structure of this article is as follows: Section 2 presents the research methodology, including an overview of the overall research framework as well as the construction, solution, and simulation design of the specific game model. Section 3 presents the results of sensitivity analysis, focusing on how the green research and pricing decisions, sales volumes, and revenues of green building material suppliers and producers respond to market demand risks under both strict and lenient environmental policies. This analysis ultimately identifies which type of environmental policy is more conducive to the green building sector's sustainable development. Section 4 compares the study findings with existing research, discussing theoretical and practical implications, as well as limitations. Section 5 concludes the article by summarizing the key insights.

## 2 Methodology

### 2.1 Research framework

**Fig. 2:**
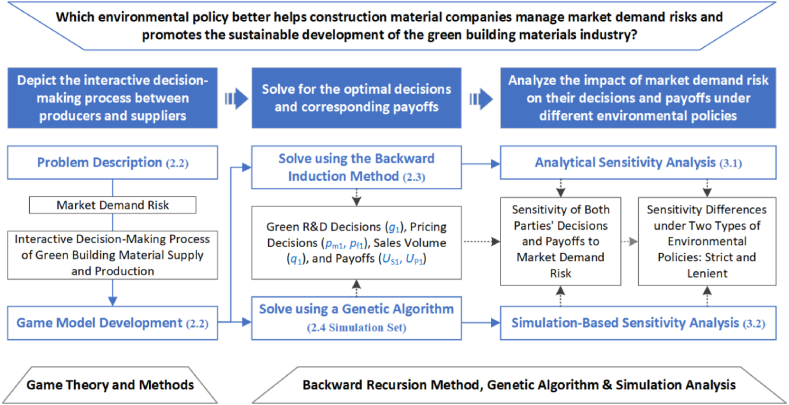
The research framework.

This study focuses on the interaction decision-making processes between building material producers and suppliers under the dual pressures of environmental policies and market demand risks. The research aims to determine which type of policy better supports companies in addressing market demand risks and promotes the sustainable development of green building materials. The study consists of three main components: the characterization and modeling of decision-making processes for producers and suppliers, the determination of optimal strategies, and the analysis of how market demand risks influence strategies and revenues under various environmental policies. The research framework is presented in Fig. 2.

First, game theory is used to characterize and model the decision-making processes between green building material producers and suppliers, aiming to identify optimal choices regarding product greenness development and pricing. Game theory serves as an effective method for analyzing the interactive decision-making of multiple stakeholders [18]. The decisions made by producers and suppliers regarding green research investment and product pricing are interactive. On one hand, suppliers are key players in green research and are responsible for pricing intermediate products. Their decisions on green research directly impact the greenness of both intermediate and final products. This greenness acts as a non-price competitive factor in the market, affecting consumer purchasing behavior and sales volume, which in turn influences producers' pricing strategies in a competitive landscape. On the other hand, producers' pricing of final products directly affects market competition and sales volume, which subsequently impacts their procurement quantities from suppliers and the suppliers' revenues. This cycle also influences the suppliers’ decisions regarding green research and intermediate product pricing. The specific modeling process detailed in Section 2.2.

Next, a combination of backward induction and genetic algorithms is employed to determine the optimal decisions for green building material suppliers and producers. In practice, the interaction between the game participants follows a clear sequential pattern: suppliers first decide on investments in greenness research and the pricing of intermediate products, and producers subsequently set the final product prices in response to market competition. When making decisions, companies must consider how current choices will affect future actions and revenues; this makes backward induction a suitable method for calculating game outcomes. However, this approach is limited to finite-step games and relies on assumptions of complete rationality and information, often yielding only local optima. In reality, the interactions between green building material producers and suppliers are ongoing and may be influenced by factors such as decision-making capabilities and information asymmetry, falling under the category of bounded rationality in an incomplete information context. To address the limitations of backward induction, this study further applies genetic algorithms, which are effective for solving multi-step or complex problems without requiring complete information or rationality from participants. These algorithms find approximate global optima through global search techniques, and their effectiveness largely depends on parameter settings. Thus, this research designs simulation parameters by integrating results from backward induction and real-world case data. This approach optimizes initial conditions for the genetic algorithm, narrows the search space, and enhances the informativeness of simulation results. Analyzing the results from both algorithms collectively contributes to the reliability of the study's findings. The backward induction solving process is detailed in Section 2.3, while the simulation setup for the genetic algorithm is outlined in Section 2.4.

Finally, using a sensitivity analysis framework combined with mathematical analysis and simulation methods, we analyze the impact of market demand risks on the decisions and profits of green building material suppliers and producers under different environmental policies. By comparative analysis, we identify which environmental policy better helps enterprises cope with market risks and is more beneficial for promoting the sustainable development of the green building material industry. First, based on practical experience, the market's sensitivity to the greenness of products usually differs under different environmental policies. Specifically, under strict environmental policies, green indicators are included in government procurement standards for building materials, signaling green cement demand to the market. At this point, the market's sensitivity to product greenness increases, even surpassing sensitivity to product prices. Under lenient environmental policies, the government procurement standards do not specify green indicators for building materials, and the market's sensitivity to product greenness will be lower than its sensitivity to product prices. Therefore, the market's relative sensitivities to product greenness and price can characterize the two environmental policy environments. Additionally, sensitivity analysis based on analytical methods reveals that both parties' decisions and profits, as they change with market risks, are influenced by the market's relative sensitivity to product greenness and price. The analysis results can determine specific parameter settings. On this basis, a genetic algorithm simulation analysis [19] is designed, setting sensitivity coefficients for product greenness and price under both strict and lenient environmental policies. The simulation then analyzes producers' and suppliers' strategies and profits as market risks change. By comparing the results under the two policies, we identify which environmental policy is more conducive to the survival of building material producers and suppliers and the development of the green building material industry.

### 2.2 Problem description and game model development

**Fig. 3:**
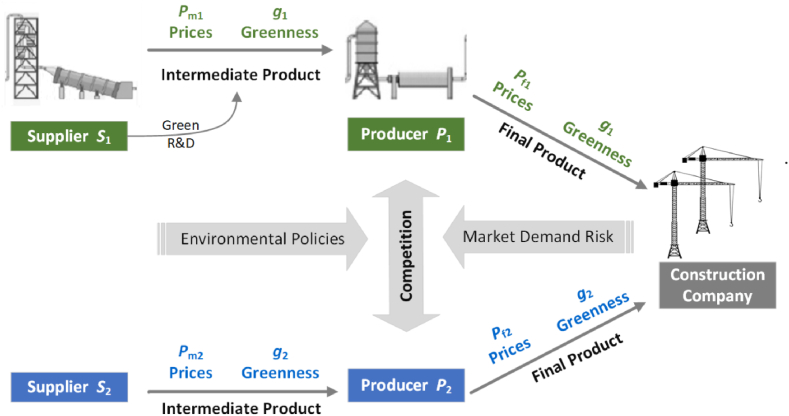
Strategic interactions between Green Building Material Producers and Suppliers.

In the cement industry, cement clinker production lines (such as those of Conch Cement) serve as upstream suppliers, and their production activities are often concentrated in resource-rich areas, while downstream cement grinding stations are distributed across the country. Taking Conch Cement, which ranks second in clinker production capacity in China, as an example, the company has established numerous cement clinker production lines in limestone-rich regions along the Yangtze River. These clinker products are then transported by water to areas with high cement demand but lacking limestone resources, where they supply local cement grinding plants. In the game model, the upstream clinker producer is represented as supplier ($S_{1}$), and the downstream cement grinding plant is represented as terminal producer ($P_{1}$). The interactive decisions in their supply cooperation include $P_{1}$’s pricing strategy for the final green building products ($p_{f1}$) and $S_{1}$’s strategy about products' greenness ($g_{1}$) and pricing strategy for intermediate products ($p_{m1}$). The relationships among the involved stakeholders and their strategies are illustrated in Fig. 3, while the relevant variable parameters are detailed in Table 1. This section constructs the game model based on the problem description and proposed assumptions.

**Table 1:** Parameters in the game model.

**Parameter**	**Description**
$p_{f1},p_{f2}$	Pricing strategy of producer $P_{1},P_{2}$, specifically the price of the final product
$p_{m1},p_{m2}$	Pricing strategy of supplier $S_{1},S_{2}$, specifically the price of the intermediate product
$g_{1},g_{2}$	Greenness of products $G_{1}$ and $G_{2}$
$q_{1}$	Sales volume of product $G_{1}$
$U_{P1}$	Profit of producer $P_{1}$
$U_{S1}$	Profit of supplier $S_{1}$
2Q	Potential demand for the final product in the regional market
r	Market demand risk(r≥0)
ε	Coefficient indicating the proportion of risk affecting market demand (ε>0)
α	Sensitivity of market demand to product price
β	Sensitivity of market demand to product greenness
μ	Cost coefficient for supplier's ($S_{1}$) green R&D

Assume that both $P_{1}$ and $S_{1}$ possess complete information, being fully aware of each other's strategies and payoff structures. Additionally, each final product consumes one unit of the intermediate product, establishing a one-to-one relationship between the final product's sales volume and the intermediate product's procurement volume [13,20].

The sales volume ($q_{1}$) of the building material product ($G_{1}$) produced by $P_{1}$ is determined by market competition. Currently, the cement industry exhibits characteristics of oligopolistic competition, where large companies, such as Lafarge Group and Holcim Group, increase their market share through mergers, restructuring, and technological innovations. In regional markets, a specific cement product competes with all other similar products simultaneously. Therefore, we can simplify the market to the competition between product $G_{1}$ and competing product $G_{2}$.

Assuming the potential demand for these products is 2Q, two theoretically homogeneous products could evenly split the market, each capturing a potential demand volume of Q. It is assumed that the potential demand for similar building materials is consistent across the market. The market demand risk, denoted as r, reflects the likelihood of demand fluctuations and adverse factors; when r>0, actual market demand ($Q_{r}$) falls short of expected demand (Q). r=0 indicates no demand risk, resulting in $Q_{r}=Q$. The relationship between $Q_{r}$ and Q is given by equation (1).

(1) $$Q_{r}=Q{∙e}^{-\varepsilon r}$$

Where, e≈2.71828 is the base of natural logarithms. In this equation, ε represents the coefficient indicating how risk levels affect market demand. This relationship draws from the literature on economic modeling of demand uncertainty, which commonly applies exponential functions to describe the diminishing impact of increasing risk on market demand (Baxter & Crucini, 1995; Kwak & Kim, 2006).

Empirical evidence and existing theoretical models suggest that actual sales volume is influenced by potential market demand, product pricing, and non-price competitive factors [21,22]. This study focuses on the product's greenness as a non-price competitive factor. $q_{1}$ is negatively correlated with the price difference between $G_{1}$ and $G_{2}$ ($p_{f1}-p_{f2}$) and positively correlated with the difference in greenness between $G_{1}$ and $G_{2}$ ($g_{1}-g_{2}$) [13], as shown in equation (2). Market sensitivity to product price and non-price indicators is denoted by α and β, respectively.

(2) $$q_{1}=Q_{r}-\alpha \left(p_{f1}-p_{f2}\right)+\beta \left(g_{1}-g_{2}\right)$$

The green research and development cost ($C_{S1}$) of supplier S₁ is a convex function of the product's greenness ($g_{1}$) [13,23], as shown in equation (3). The parameter μ represents the cost coefficient for $S_{1}$’s green R&D efforts. μ must not take an excessively high value [20,24] as a high R&D cost coefficient may deter the supplier from engaging in research and development activities. This product is considered development-intensive, meaning its R&D costs are fixed and do not vary with production volume, unlike marginal cost-intensive products. The supplier's R&D efforts are assumed to not adversely affect the quality, capacity, or stability of downstream producers' production processes [13].

(3) $$C_{S1}=\mu {g_{1}}^{2}/2$$

Additionally, producer $P_{1}$ and supplier $S_{1}$ incur production costs for the final ($D_{P1}$) and intermediate ($D_{S1}$) products, respectively. These costs are variable and depend on the quantity produced. To simplify calculations, assume $D_{P1}=D_{S1}=0$. Since this study does not involve the supplier's quantity decision, this simplification does not affect the qualitative research outcomes derived from the model, while also enhancing the clarity of representation of product technology levels [19].

Based on this, we can establish the expected profit functions for $P_{1}$ and $S_{1}$ in their supply cooperation ($U_{P1}$ and $U_{S1}$), as shown in equations (4), (5).

(4) $$U_{P1}=\left(p_{f1}-p_{m1}\right)\left[Qe^{-\varepsilon r}-\alpha \left(p_{f1}-p_{f2}\right)+\beta \left(g_{1}-g_{2}\right)\right]$$

(5) $$U_{S1}=p_{m1}\left[Qe^{-\varepsilon r}-\alpha \left(p_{f1}-p_{f2}\right)+\beta \left(g_{1}-g_{2}\right)\right]-\mu {g_{1}}^{2}/2$$

### 2.3 Game model solution

Using the backward induction method, we sequentially determine the optimal solutions for the final product price, intermediate product price, and green research investment.

First, we examine the second-order partial derivative of the profit function $U_{P1}$ with respect to $p_{f1}$, ${d^{2}U}_{P1}/d{p_{f1}}^{2}=-2\alpha <0$, which indicates that $U_{P1}$ is a concave function in terms of $p_{f1}$. Setting the first derivative ${dU}_{P1}/dp_{f1}=0$ allows us to find the optimal solution ${p_{f1}}^{*}$, as shown in equation (7).

Next, we substitute ${p_{f1}}^{*}$ into the function $U_{S1}$ to evaluate the second-order partial derivatives with respect to $p_{m1}$ and $g_{1}$. We find that ${d^{2}U}_{S1}/d{p_{m1}}^{2}=-2\alpha /3<0$ and ${d^{2}U}_{S1}/d{g_{1}}^{2}=-\mu <0$. The Hessian matrix, as shown in equation (6), is given by $\left|H\right|=\left(6\alpha \mu -\beta^{2}\right)/9>0$, confirming that $U_{S1}$ is a concave function with respect to $p_{m1}$ and $g_{1}$. By setting the first derivatives of $U_{S1}$ with respect to $p_{m1}$ and $g_{1}$ equal to zero, we derive the optimal solutions ${p_{m}}^{*}$ and ${g_{1}}^{*}$, as shown in equations (8), (9).

(6) $$H=\left(\begin{array}{cc} -\frac{2\alpha }{3} & \frac{\beta }{3}\\ \frac{\beta }{3} & -\mu \end{array}\right)$$

(7) $${p_{f1}}^{*}=\frac{3\mu \left(Qe^{-\varepsilon r}-\beta g_{2}+\alpha p_{f2}\right)}{4\alpha \mu -\beta^{2}}$$

(8) $${p_{m1}}^{*}=\frac{2\mu \left(Qe^{-\varepsilon r}-\beta g_{2}+\alpha p_{f2}\right)}{4\alpha \mu -\beta^{2}}$$

(9) $${g_{1}}^{*}=\frac{\beta \left(Qe^{-\varepsilon r}-\beta g_{2}+\alpha p_{f2}\right)}{4\alpha \mu -\beta^{2}}$$

Next, we get ${q_{1}}^{*}$, as shown in equation (10), by substituting ${p_{f1}}^{*},{p_{m}}^{*}$, and ${g_{1}}^{*}$ into the final product sales volume function $q_{1}$. We then impose the condition $\frac{\alpha \mu \left(Qe^{-\varepsilon r}-\beta g_{2}+\alpha p_{f2}\right)}{4\alpha \mu -\beta^{2}}\geq 0$ to ensure that the sales volume remains non-negative. This aligns with similar assumptions found in various studies [[25], [26], [27]]. The parameter constraints to satisfy this condition are as follows: $4\alpha \mu -\beta^{2}\neq 0$. When $4\alpha \mu -\beta^{2}>0$, the condition simplifies to $Qe^{-\varepsilon r}-\beta g_{2}+\alpha p_{f2}\geq 0$; conversely, if $4\alpha \mu -\beta^{2}<0$, then $Qe^{-\varepsilon r}-\beta g_{2}+\alpha p_{f2}\leq 0$. Under these conditions, ${p_{f1}}^{*}\geq 0,{p_{m}}^{*}\geq 0,{g_{1}}^{*}\geq 0$.

(10) $${q_{1}}^{*}=\frac{\alpha \mu \left(Qe^{-\varepsilon r}-\beta g_{2}+\alpha p_{f2}\right)}{4\alpha \mu -\beta^{2}}$$

Finally, we substitute ${p_{f1}}^{*},{p_{m}}^{*}$, and ${g_{1}}^{*}$ into the profit functions $U_{P1}$ and $U_{S1}$ to obtain the profits ${U_{P1}}^{*}$ and ${U_{S1}}^{*}$ for the producer and supplier under their optimal strategies, as shown in equations (11), (12).

(11) $${U_{P1}}^{*}=\frac{\mu^{2}{\left(Qe^{-\varepsilon r}-\beta g_{2}+\alpha p_{f2}\right)}^{2}}{{\left(4\alpha \mu -\beta^{2}\right)}^{2}}$$

(12) $${U_{S1}}^{*}=\frac{\mu {\left(Qe^{-\varepsilon r}-\beta g_{2}+\alpha p_{f2}\right)}^{2}}{2\left(4\alpha \mu -\beta^{2}\right)}$$

### 2.4 Simulation set

This study aims to use genetic algorithms to simulate the interactive decision-making processes between green building material suppliers and producers regarding green research investments, pricing of intermediate and final products. By analyzing the sensitivity of these decisions, sales volumes, and profits to market demand risks under strict or lenient environmental policies, we seek to identify which policies better support building material companies in managing market risks and promote the sustainable development of the green building material industry.

Using market data for cement products and clinker prices, we set the simulation parameters as shown in Table 2. Records of the recent three years from the Chinese Cement Website [5] indicate that cement prices fluctuate between 310 and 650 yuan per ton, while clinker prices range from 270 to 510 yuan per ton. The required amount of clinker for different types of cement varies significantly, ranging from 0.285 to 0.95 tons. The most demanded product, ordinary Portland cement, requires approximately 0.8–0.9 tons of clinker per ton produced. Therefore, we set the clinker costs between 216 and 459 yuan.

**Table 2:** Parameter values for simulation analysis.

**Parameters**	**Values (Units)**	**Parameters**	**Values**	**Parameters**	**Values**
$p_{f1},p_{f2}$	0.3–0.7 (1000 yuan)	r	0∼5	β	0.1 or 5.5
$p_{m1}$	0.2–0.5 (1000 yuan)	ε	1	Population Size	200
$g_{1},g_{2}$	0.3–0.7 (1000)	α	1	Crossover Rate	0.8
Q	1	μ	1	Mutation Rate	0.01

To simplify calculations and ensure comparability, we adjust the price units to thousands of yuan. The price ranges for cement ($p_{f1},p_{f2}$) are set at 0.3–0.7, and the price of clinker ($p_{m1},p_{m2}$) is set at 0.2–0.5. For comparability with green product metrics, we set the range for greenness at 0.3–0.7, with a potential market demand (Q) of 1. We assume the risk coefficient (ε) affecting market demand is 1, and when εr=5, $e^{-\varepsilon r}\approx 0$. Thus, we set the market demand risk (r) to range from 0 to 5. The sensitivity of market demand to product prices (α) and the supplier's green research cost coefficient (μ) are both set to 1. Based on our analytical results regarding optimal strategies and profit sensitivity to market demand risk, we designate the sensitivity coefficients for product greenness as 0.1 and 5.5, representing scenarios of lenient ($4\alpha \mu -\beta^{2}>0$) and strict ($4\alpha \mu -\beta^{2}<0$) environmental policies, respectively.

**Fig. 4:**
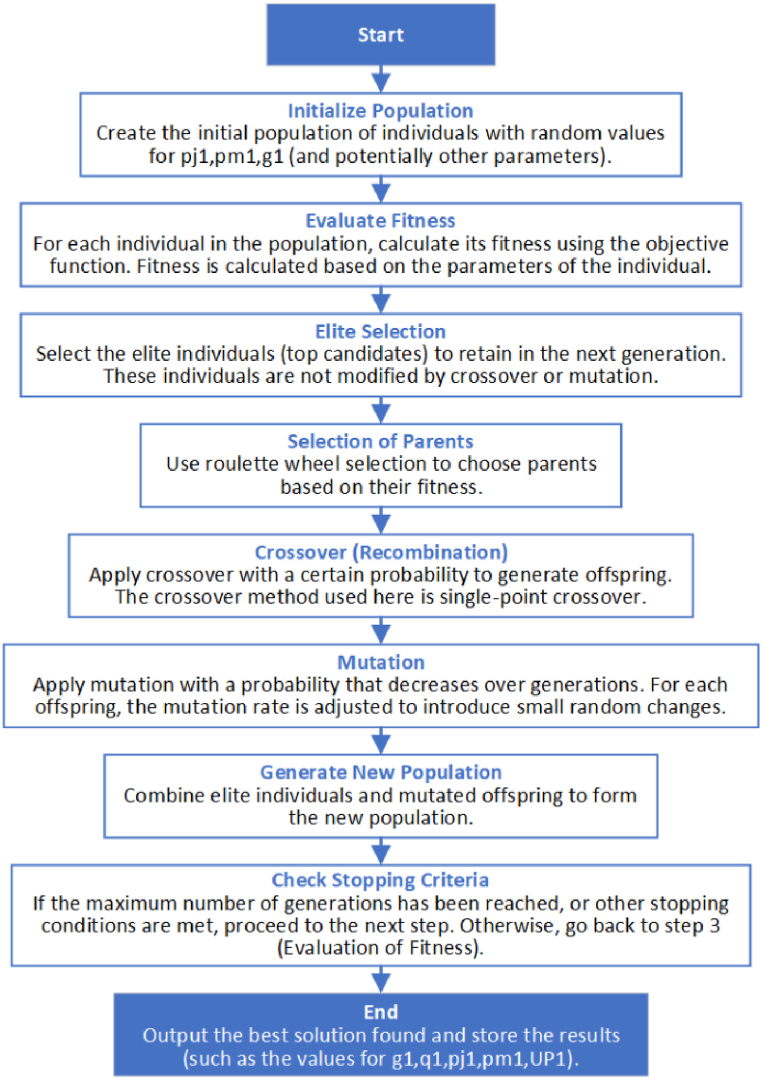
Genetic algorithm flowchart.

We implemented the genetic algorithm using MATLAB in the LinRx environment and conducted large-scale numerical simulations on the JH Innovation Software Version 5.3.1 supercomputing platform, with the genetic algorithm flowchart shown in Fig. 4 and code provided in appendix B. The genetic algorithm was set with a population size of 200, a crossover rate of 0.8, a mutation rate of 0.01, and a total of 100 iterations. Additionally, we repeated the experiments 100 times using Monte Carlo simulation.

## 3 Sensitivity analysis results

### 3.1 Analytical analysis results

This section analyzes the sensitivity of the optimal strategies $\left({p_{f1}}^{*},{p_{m}}^{*},{g_{1}}^{*}\right)$, sales $\left({q_{1}}^{*}\right)$, and profits $\left({U_{P1}}^{*},{U_{S1}}^{*}\right)$ of green building material manufacturers and suppliers to the market sensitivity coefficients for product price and greenness (α,β) and market demand risk (r) using derivative methods. In a relatively short time window, the probability of significant changes in the green R&D cost coefficient (μ) due to technological advancements is low, and this section does not conduct a sensitivity analysis on μ. However, adjustments in environmental policies can lead to substantial changes in the sensitivity of market demand for building materials to product prices and greenness. Therefore, sensitivity must be analyzed with respect to α and β. The analysis process includes two steps. First, we compute the first-order partial derivatives of ${p_{f1}}^{*},{p_{m}}^{*},{g_{1}}^{*}{{,{q_{1}}^{*},U}_{P1}}^{*},{{\;U}_{S1}}^{*}$ with respect to α,β,r, as shown in Appendix A. Then, by analyzing the sign of the partial derivatives, we determine the direction in which ${p_{f1}}^{*},{p_{m}}^{*},{g_{1}}^{*}{{,{q_{1}}^{*},U}_{P1}}^{*},{{\;U}_{S1}}^{*}$ change with respect to α,β,r; if the derivative is greater than 0, they are positively correlated, and if less than 0, they are negatively correlated. We conduct the analytical analysis under the condition that $4\alpha \mu -\beta^{2}\neq 0$ and ${q_{1}}^{*}\geq 0$ (i.e., $4\alpha \mu -\beta^{2}$ and $Qe^{-\varepsilon r}-\beta g_{2}+\alpha p_{f2}$ have the same positive or negative sign to ensure that the sales are not negative, which has already been proven in Section 2.3).

**Fig. 5:**
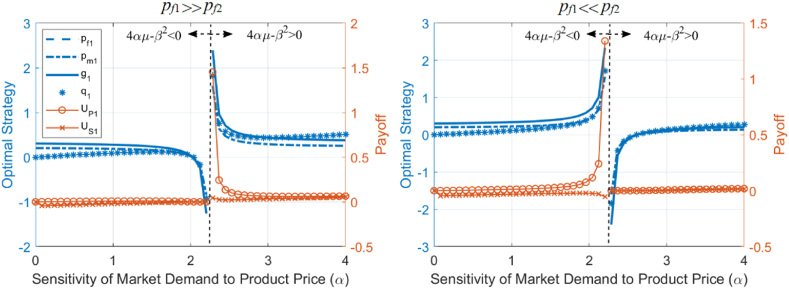
Analytical results of optimal strategies, sales volume, and profits sensitivity to the sensitivity of market demand to product price.

The analysis reveals that the optimal strategies optimal strategies $\left({p_{f1}}^{*},{p_{m}}^{*},{g_{1}}^{*}\right)$, sales $\left({q_{1}}^{*}\right)$, and profits $\left({U_{P1}}^{*},{U_{S1}}^{*}\right)$ are influenced by the relative size of ${p_{f1}}^{*}$ and $p_{f2}$ as α changes. As shown in Fig. 5, different types of blue curves represent ${p_{f1}}^{*},{p_{m}}^{*},{g_{1}}^{*},{q_{1}}^{*}$ (corresponding to the left vertical axis), and the red curves with different labels represent ${U_{P1}}^{*},{U_{S1}}^{*}$ (corresponding to the right vertical axis), with the horizontal axis representing α. In the left panel of Fig. 5, the parameter values satisfy ${p_{f1}}^{*}\gg p_{f2}$, where $2{p_{f1}}^{*}>{3p}_{f2}$ and ${{\beta^{2}p}_{f1}}^{*}>{3\alpha \mu p}_{f2}$; in the right panel, the parameter values satisfy ${p_{f1}}^{*}\ll p_{f2}$, where ${{4p}_{f1}}^{*}<{3p}_{f2}$ and ${{\beta^{2}p}_{f1}}^{*}<3\alpha \mu p_{f2}$. Based on the derivative formulas and Fig. 5, we observe the following: (1) when ${{4p}_{f1}}^{*}>{3p}_{f2}$, the first derivative of ${p_{f1}}^{*},{p_{m}}^{*},{g_{1}}^{*}{{,U}_{P1}}^{*}$ with respect to α is less than 0, and they decrease as α increases; when ${{4p}_{f1}}^{*}<{3p}_{f2}$, they increase as α increases; (2) when ${{\beta^{2}p}_{f1}}^{*}>{3\alpha \mu p}_{f2}$, the first derivative of ${q_{1}}^{*}$ with respect to α is less than 0, meaning ${q_{1}}^{*}$ decreases as α increases; when ${{\beta^{2}p}_{f1}}^{*}<{3\alpha \mu p}_{f2}$, ${q_{1}}^{*}$ increases as α increases; (3) when ${2p_{f1}}^{*}-3p_{f2}$ and $4\alpha \mu -\beta^{2}$ have the same sign, the first derivative of ${U_{S1}}^{*}$ with respect to α is less than 0, and ${U_{S1}}^{*}$ decreases as α increases; when they have opposite signs, ${U_{S1}}^{*}$ increases as α increases.

This analysis reflects the impact of the price sensitivity of market demand on the optimal strategies, sales, and profits of building material manufacturers and suppliers, modulated by the relative prices of the product and competing products. When the product price is higher than the competitor's price, an increase in market demand's price sensitivity leads to a decrease in product price, green attributes, sales volume, and manufacturer profits; when the product price is lower than the competitor's price, an increase in market demand's price sensitivity leads to an increase in product price, green attributes, sales volume, and manufacturer profits.

**Fig. 6:**
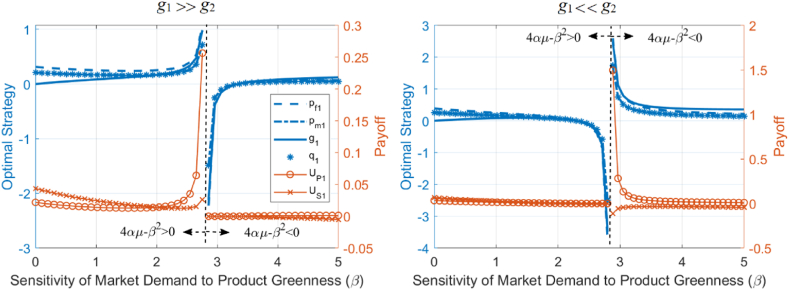
Analytical results of optimal strategies, sales volume, and profits sensitivity to the sensitivity of market demand to product greenness.

Additionally, the directions of change for ${p_{f1}}^{*},{p_{m}}^{*},{g_{1}}^{*}{{,{q_{1}}^{*},U}_{P1}}^{*},{U_{S1}}^{*}$ with respect to β are influenced by the relative sizes of ${g_{1}}^{*}$ and $g_{2}$. In Fig. 6, the parameter values in the left panel satisfy ${g_{1}}^{*}\gg g_{2}$, where ${\left({4\alpha \mu +\beta }^{2}\right)g_{1}}^{*}>\beta^{2}g_{2}$; the parameter values in the right panel satisfy ${g_{1}}^{*}\ll g_{2}$, where $2{g_{1}}^{*}<g_{2}$ and ${\left({4\alpha \mu +\beta }^{2}\right)g_{1}}^{*}<\beta^{2}g_{2}$. Based on the derivative formulas and Fig. 6, we observe the following: (1) when $2{g_{1}}^{*}>g_{2}$, the first derivatives of ${{\;p}_{f1}}^{*},{p_{m}}^{*},{q_{1}}^{*}{{,U}_{P1}}^{*}$ with respect to β are all greater than 0, indicating that ${p_{f1}}^{*},{p_{m}}^{*},{q_{1}}^{*}{{,U}_{P1}}^{*}$ increase as β increases; when $2{g_{1}}^{*}<g_{2}$, ${p_{f1}}^{*},{p_{m}}^{*},{q_{1}}^{*}{{,U}_{P1}}^{*}$ decrease as β increases; (2) when ${\left({4\alpha \mu +\beta }^{2}\right)g_{1}}^{*}>\beta^{2}g_{2}$, the first derivative of ${g_{1}}^{*}$ with respect to β is greater than 0, indicating that ${g_{1}}^{*}$ increases as β increases; when ${\left({4\alpha \mu +\beta }^{2}\right)g_{1}}^{*}<\beta^{2}g_{2}$, ${g_{1}}^{*}$ decreases as β increases; (3) When ${g_{1}}^{*}-g_{2}$ and $4\alpha \mu -\beta^{2}$ have the same sign, the first derivative of ${U_{S1}}^{*}$ with respect to β is greater than 0, and ${U_{S1}}^{*}$ increases as β increases; when they have opposite signs, ${U_{S1}}^{*}$ decreases as β increases.

This analysis demonstrates that the market's sensitivity to a product's greenness affects the optimal strategies, sales, and profits of building material producers and suppliers. This effect is moderated by the product's relative greenness compared to its competing products. When the product's greenness is higher than that of competing products, an increase in market sensitivity leads to higher product prices, greenness, sales, and producer profits. Conversely, when the product's greenness is lower than that of competing products, an increase in market sensitivity leads to lower product prices, greenness, sales, and producer profits.

**Fig. 7:**
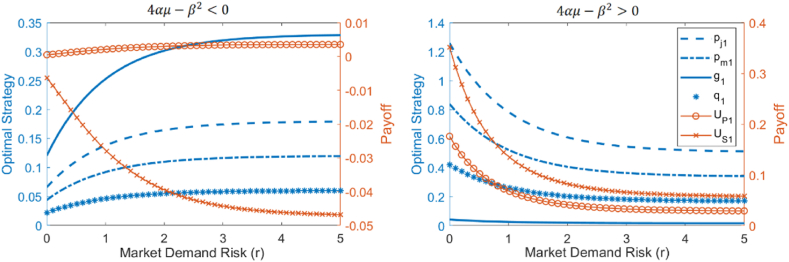
Analytical results of optimal strategies, sales volume, and profits sensitivity to market demand risk.

Additionally, ${U_{S1}}^{*}$ is negatively correlated with both r. The direction of change for ${p_{f1}}^{*},{p_{m}}^{*},{g_{1}}^{*}{{,{q_{1}}^{*},U}_{P1}}^{*}$ with respect to r is influenced by the sign of $4\alpha \mu -\beta^{2}$. In Fig. 7, the left panel corresponds to the case where $4\alpha \mu -\beta^{2}<0$, while the right panel corresponds to $4\alpha \mu -\beta^{2}>0$. Based on the derivative formulas and Fig. 7, we observe the following: (1) the first derivative of ${U_{S1}}^{*}$ with respect to r is always less than 0, indicating that ${U_{S1}}^{*}$ always decreases as r increases; (2) when $4\alpha \mu -\beta^{2}<0$, the first derivatives of ${p_{f1}}^{*},{p_{m}}^{*},{g_{1}}^{*}{{,{q_{1}}^{*},U}_{P1}}^{*}$ with respect to r are all greater than 0, indicating that ${p_{f1}}^{*},{p_{m}}^{*},{g_{1}}^{*}{{,{q_{1}}^{*},U}_{P1}}^{*}$ increase as r increases; when $4\alpha \mu -\beta^{2}>0$, the first derivatives of ${p_{f1}}^{*},{p_{m}}^{*},{g_{1}}^{*}{{,{q_{1}}^{*},U}_{P1}}^{*}$ with respect to r are all less than 0, meaning they decrease as r increases.

profits of building material producers and suppliers, which is influenced by the market's relative sensitivity to product prices and greenness. When market demand is far more sensitive to product prices than to greenness, an increase in market risk will lead to higher product prices, greenness, sales, and producer profits. Conversely, when the market demand is far more sensitive to the product's greenness than to its price, an increase in market risk will lead to lower product prices, greenness, sales, and producer profits.

Further elaborate on the above analysis results in the context of environmental policy. Adjustments in environmental policies can lead to substantial changes in the sensitivity of market demand for building materials to product prices and greenness. Specifically, under strict environmental policies, green indicators are incorporated into government procurement standards, resulting in the market being much more sensitive to the greenness of building materials than to their prices (β≫α), corresponding to $4\alpha \mu -\beta^{2}<0$. The above analytical results indicate that the impact of market demand risk (r) on decisions, sales, and profits (such as ${p_{f1}}^{*},{p_{m}}^{*},{g_{1}}^{*},{q_{1}}^{*},{U_{P1}}^{*}$) differs under the two environmental policies and needs to be analyzed separately. Additionally, the market's sensitivity to product prices and greenness (α,β) directly affects ${p_{f1}}^{*},{p_{m}}^{*},{g_{1}}^{*},{q_{1}}^{*},{U_{P1}}^{*}$ and this effect is influenced by the relative size of the product price and greenness $\left({p_{f1}}^{*},{g_{1}}^{*}\right)$ compared to that of competing products $\left(p_{f2},g_{2}\right)$. Therefore, in subsequent simulations analyzing the impact of market risk under different environmental policies, the varying values of competing products' prices and greenness must also be considered.

### 3.2 Simulation analysis results

This section analyzes the impact of market demand risk on product greenness ($g_{1}$), prices ($p_{m1},p_{f1}$), sales volume ($q_{1}$), and the profits ($U_{P1},U_{S1}$) of green building material manufacturers and suppliers through a genetic algorithm simulation approach.

**Fig. 8:**
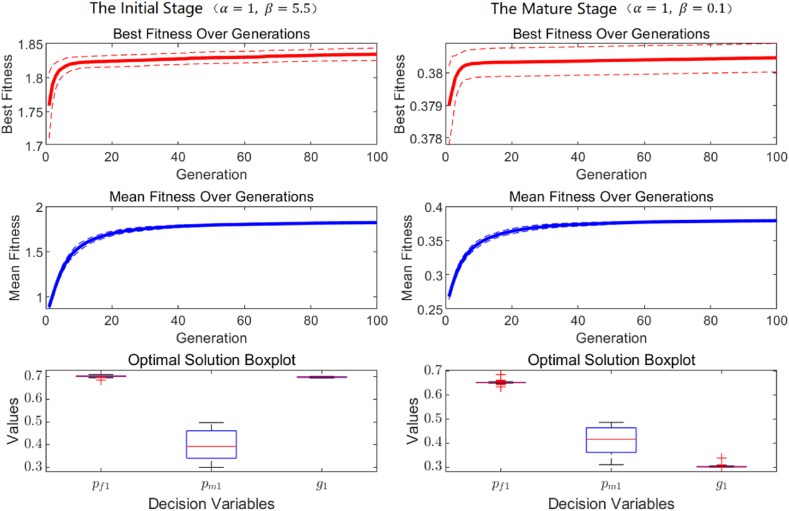
Fitness and optimal solutions from genetic algorithm.

Fig. 8 presents the simulation results of the genetic algorithm, displaying, from top to bottom, the change curves for the best fitness mean and standard deviation, the average fitness means and standard deviation, and a box plot showing the mean and range of optimal results. The results indicate that over 100 iterations, both the best and average fitness values rapidly increased and stabilized after several iterations. This trend demonstrates that the genetic algorithm effectively converges within a short iteration period and possesses strong global search capabilities. The stability of the fitness values further confirms the genetic algorithm's reliability and effectiveness in addressing this type of optimization problem. Therefore, the genetic algorithm provides efficient and stable solutions for the issues presented in this study.

**Fig. 9:**
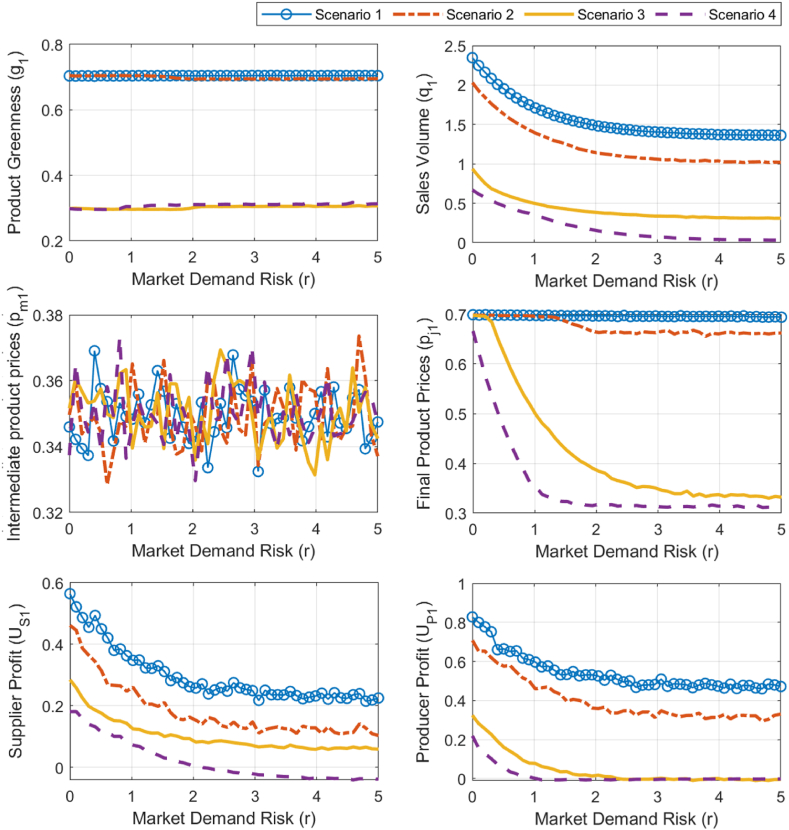
Simulation analysis of optimal strategies, sales volume, and profits sensitivity to market demand risk under scenarios 1–4.

**Fig. 10:**
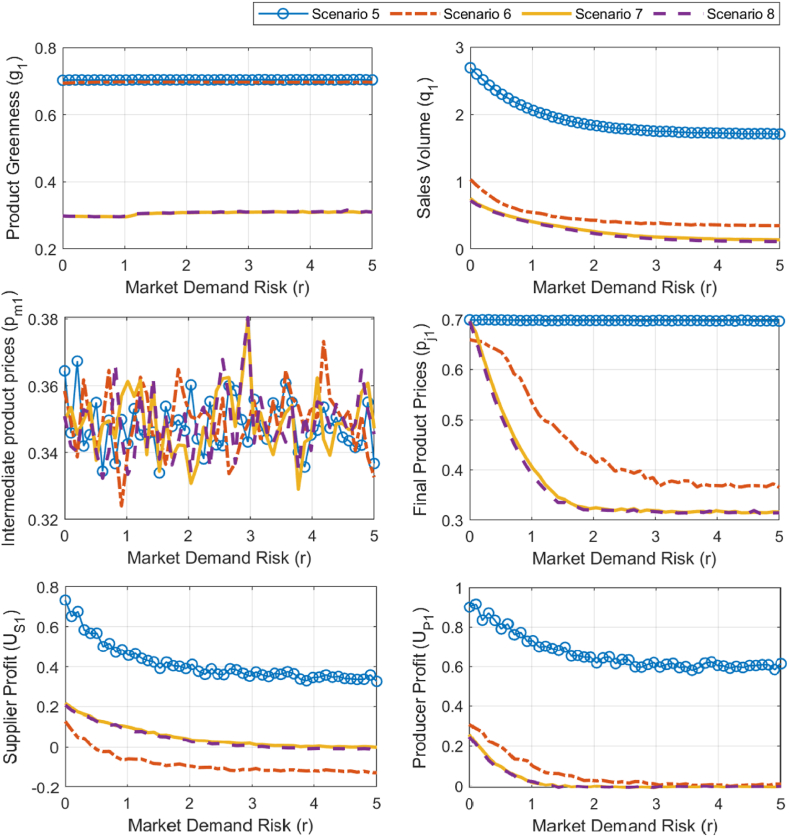
Simulation analysis of optimal strategies, sales volume, and profits sensitivity to market demand risk under scenarios 5–8.

Based on the analysis results in Section 3.1, under two different environmental policies, the influence of competing product greenness ($g_{2}$) and pricing ($p_{f2}$) on the simulation results is considered further, allowing for a breakdown of scenarios. Initially, with a fixed competing product greenness ($g_{2}=0.45$), scenarios are differentiated based on the competing product's price. Under strict environmental policy α=0.1), Scenario 1 ($p_{f2}=0.65$) and Scenario 2 ($p_{f2}=0.35$) are defined, while under lenient environmental policy (α=3.5), Scenario 3 ($p_{f2}=0.65$) and Scenario 4 ($p_{f2}=0.35$) are differentiated. The corresponding simulation results are shown in Fig. 9. Subsequently, with a fixed competing product price ($p_{f2}=0.45$), scenarios are distinguished based on competing product greenness. Under strict environmental policy (β=5.5), Scenario 5 ($g_{2}=0.35$) and Scenario 6 ($g_{2}=0.65$) are differentiated, and under lenient environmental policy (β=0.1), Scenario 7 ($g_{2}=0.35$) and Scenario 8 ($g_{2}=0.65$) are defined. The simulation results for these scenarios are illustrated in Fig. 10. By differentiating between these scenarios, the study provides a comprehensive assessment of the potential impact of market demand risk on decision-making and profit performance for building material enterprises. Analyzing the results from Fig. 9, Fig. 10 reveals the following.

First, strict environmental policies are more effective in mitigating the adverse impact of market demand risk (r) on green research and development and production in the building materials sector and maintaining a higher level of product greenness ($g_{1}$). Specifically, under strict environmental policies (scenarios 1, 2, 5, and 6), $g_{1}$ remains consistently high at around 0.7 and does not change significantly with r. Under lenient environmental policies (scenarios 3, 4, 7, and 8), $g_{1}$ sharply increases as r increases, and then stabilizes around 0.3, always remaining lower than $g_{1}$ under strict environmental policies.

Moreover, strict environmental policies help reduce the negative impact of market demand risk (r) on the final product price of green building materials ($p_{f1}$), allowing it to remain at a higher level; conversely, we observe no significant difference between the two policies regarding the maintenance of intermediate product prices ($p_{m1}$). Specifically, under strict policies, when the greenness of competing products is low (scenarios 1, 2, and 5, $g_{2}\leq 4.5$), $p_{f1}$ remains at a high level, around 0.7, as r increases. However, when the greenness of competing products is high (scenario 6, $g_{2}=6.5$), $p_{f1}$ decreases sharply with r at first and then stabilizes, with the stable value above 0.35. Under lenient policies (scenarios 3, 4, and 7, 8), $p_{f1}$ first decreases sharply as r increases and then stabilizes, with the stable value lower than 0.35, and is always lower than $p_{f1}$ under strict environmental policies. Under both environmental policies, $p_{m1}$ fluctuates continuously between 0.33 and 0.37 as r increases, and we observe no significant difference in the range of $p_{m1}$ values between the two policy contexts.

Additionally, in both policy contexts, the sales volume of green building materials ($q_{1}$) and the profits of production and supply companies ($U_{P1}$ and $U_{S1}$) decline as market demand risk (r) increases. Generally, strict environmental policies enable higher product sales and company profits. Specifically, $q_{1}$, $U_{P1}$, and $U_{S1}$ all decrease significantly with rising r before stabilizing. The values of $q_{1}$ and $U_{P1}$ under strict policies (scenarios 1, 2, and 5, 6) are consistently higher than those under lenient policies (scenarios 3, 4, and 7, 8). Additionally, when the greenness of competing products ($g_{2}$) is lower (scenarios 1, 2, and 5), $q_{1}$ and $U_{P1}$ are even higher. The relative size of $U_{S1}$ across both policies is influenced by the greenness $g_{2}$ of competing products. When the greenness is low (${\text{scenarios}\;1-4\;\text{and}\;5,7,g}_{2}\leq 4\text{.}$), $U_{S1}$ is higher under strict policies, whereas when greenness is high (scenarios 6, 8, $g_{2}=6.5$), $U_{S1}$ is higher under lenient policies.

The above simulation analysis reveals that, in situations where market demand risk increases, strict environmental policies are more beneficial for building material companies as they help enhance product green degree, sales, and corporate profits, thereby promoting the sustainable development of the green building material industry. A real-world example is that of October 2020, when the central government first explicitly proposed specific requirements for the procurement of green building materials. Further, in 2022, despite a continued decline in market demand, Conch Cement invested 5 billion yuan in energy-saving and emission-reduction projects. This illustrates that under the push of strict environmental policies, Conch Cement chose to invest more in green production technologies (such as more efficient energy use and production processes with less pollution), thus improving the green degree of its cement products; this is consistent with the conclusions of the simulation analysis. Moreover, strict environmental policies have raised the green degree acceptance threshold in major markets for cement, while also giving green cement more bargaining power compared to ordinary cement. Furthermore, improving the green degree of products has helped Conch Cement and its downstream cement grinding plants gain a greater sales advantage, which has resulted in higher profits.

### 3.3 Comparison analysis

Both the sensitivity analysis results based on the reverse recursion method and the sensitivity simulation results based on the genetic algorithm indicate that strict environmental policies are more beneficial for building material companies in responding to market risks and promoting the sustainable development of the green building material industry. Both analysis methods show that under different environmental policies, the direction of change in the final product's green degree and price varies with market demand risk. Strict environmental policies are more conducive to promoting the increase of the final product's green degree and price. Specifically, under strict environmental policies $\left(4\alpha \mu -\beta^{2}<0\right)$, both the product's green degree and price increase with the rise in market demand risk, whereas under lenient environmental policies ($4\alpha \mu -\beta^{2}>0)$, both decrease with the increase in market demand risk. The simulation results show that as market risk increases, the product's green degree under strict environmental policies stabilizes around 0.7, which is higher than the stable level of 0.3 under lenient environmental policies. Additionally, the final product price under strict environmental policies remains consistently higher than the optimal price under lenient environmental policies.

However, the findings diverge regarding intermediate product prices, final product sales, and manufacturer profits under strict policies. Specifically, the analytical results show that under strict environmental policies $\left(4\alpha \mu -\beta^{2}<0\right)$, the intermediate product price, final product sales, and producer profits all increase with the rise in market demand risk. However, the simulation results show that final product sales and producer profits both decrease, while the intermediate product price fluctuates between 0.33 and 0.37. Further comparative analysis that considers the characteristics of the methods and real-world experience must be conducted.

Theoretically, the results from the backward recursive method are more precise and stable, yet they yield local optimal solutions and are highly dependent on model assumptions, which makes them susceptible to the limitations of simplified game models. In contrast, genetic algorithms serve as global optimization techniques that can identify solutions close to the global optimum; however, the precision of their results is influenced by factors such as population size and the number of iterations and thus lacks guaranteed accuracy. The aim of sensitivity analysis is to determine which environmental policy better assists building material companies in addressing market demand risks, focusing on comparative outcomes rather than precise solutions. Given the theoretical characteristics of both methods, the preference is for the results obtained from simulation analysis.

In light of real-world experiences, the recent downturn in China's real estate sector has led to substantial increases in market demand risks. For instance, cement prices have plummeted from 650 yuan per ton to 320 yuan, resulting in noticeable declines in sales volumes and profits for related enterprises. The analytical findings obtained through the backward recursive method suggest that under strict environmental policies, product sales and prices increase with market demand risk contradict common sense and real-world observations, which prompts their exclusion from consideration. Conversely, the simulation results from genetic algorithms indicate that as market demand risk rises, strict environmental policies help maintain high levels of product greenness and final product prices, while intermediate prices fluctuate, and both sales volumes and profits decline. These findings align more closely with the observed realities.

## 4 Discussion

### 4.1 Main findings

This study focuses on the increasing market demand risk in the building materials sector and compares the changes in product green degree, price, sales, and producer and supplier profits under different environmental policies and varying pricing or green degree levels of competing products. The main findings reveal that strict environmental policies are more effective than lenient policies in addressing increasing market demand risk while simultaneously improving the green degree of building materials and increasing enterprise profits. Specifically, by comparing scenarios 1, 2, 5, 6 with scenarios 3, 4, 7, 8, we draw the following conclusions.

1) As market demand risk continues to rise, the green degree of building materials under strict environmental policies (around 0.7) remains higher than that under lenient environmental policies (around 0.3). This suggests that strict environmental policies are more conducive to enhancing green investment and product green degree in the face of high market demand risks.
2) During the process of increasing market demand risk, the intermediate product price shows no significant difference between the two policies, fluctuating between 0.33 and 0.37. However, the final product price (around 0.7) and sales (greater than 1) under strict environmental policies are higher than under lenient policies (final product price around 0.3, and sales less than 1). This leads to higher profit levels for producers and suppliers under strict environmental policies.
3) Additionally, by comparing scenarios 5 and 6, we find that when the green degree of competing products is low, strict environmental policies show a more prominent advantage in maintaining green building material sales, prices, and enterprise profits.

### 4.2 Literature comparison and theoretical contributions

In comparison to the existing literature, this research provides additional evidence and perspectives on the topic. This study highlights that strict environmental policies are more conducive to the green building sector's sustainable development under market demand risks. Consistent with this finding, Siyi Zhang et al. [28] noted that command policies may inhibit green economic efficiency in the short term, but their long-term effects enhance this efficiency. Additionally, existing research conclusions have provided us with a theoretical framework to explain the results of this study, and in turn, our findings also contribute evidence to this theoretical framework. Based on data from 276 cities in China between 2003 and 2013, a study [7] demonstrated that environmental regulation has a U-shaped overall impact on green innovation. This suggests that the Porter hypothesis and crowding out theory are not in conflict but rather offer theoretical explanations for different stages of local responses to environmental regulation. Specifically, the effects of environmental regulation on green innovation are varied, exhibiting patterns that include enhancing, stagnating, undermining, U-shaped, and inverted U-shaped responses. Our findings in this support this theoretical framework by contributing evidence that the impact of environmental regulation on green research and production exhibits diverse patterns influenced by specific contexts. The market demand risk for green building materials discussed in this paper is one such context, where strict environmental policies have an “enhancing” effect on green research and production.

Compared to the existing literature, this study not only provides evidence of the cement industry's response to environmental policies under market demand risk, but it also extends the comparative analysis to other green building materials industries. In the study, strict environmental policies have significantly different impacts on various green building material industries. Specifically, the cement industry, as a traditional high-energy consumption sector, exhibits stronger green technology innovation and market adaptability when faced with strict environmental policies. However, industries such as green building panels or green insulation materials—albeit also incentivized by policies—tend to focus more on material substitution and optimization of production processes to enhance their green degree [29,30]. This difference indicates that industries exhibit varied pathways in terms of technological progress, green degree enhancement, and market performance when responding to environmental policies. Further analysis shows that the cement industry and other green building material industries also display different trends in addressing market demand risks. The demand fluctuations in the cement industry are more significantly influenced by macroeconomic factors, and therefore, its strategies mainly focus on adjusting prices and production scales [31]. In contrast, the demand for other green building materials, such as green wall materials or solar heating systems, is more influenced by policies and consumer behavior [32], leading to a more diversified strategy for managing market risks. Overall, the impact of strict environmental policies on the cement industry and other green building material industries differs and is closely related to each industry's technical requirements, market structure, and policy incentive mechanisms.

Moreover, this study significantly supplements the existing literature by focusing on scenarios where market demand risks and environmental policies are both at play. Previous research has mostly discussed the impacts of market demand risks or environmental policies on green building material enterprises separately, without delving into the complexity of their combined effects. Additionally, while some studies have examined the influence of environmental policies on corporate green behaviors, few have explored how different types of environmental policies affect corporate strategies and sustainable development against the backdrop of market demand risks. Our findings highlight that strict environmental policies are more effective in promoting green research and production—particularly under high market demand risks—and help firms maintain competitiveness. This perspective not only fills a research gap but also offers targeted recommendations for managerial practice, urging policymakers to consider the impact of market demand risks when designing environmental policies, thereby providing stronger support for the sustainable development of green building material enterprises.

The theoretical and practical significance of this study is reflected in several aspects. First, from a theoretical perspective, this research fills a gap by examining how market demand risks and environmental policies jointly influence the sustainable development of green building material enterprises. Previous literature has often focused on the impact of single factors, lacking a thorough analysis of their interplay. Our findings demonstrate that strict environmental policies enhance the sustainable development of green building materials enterprise and industry in environments with high market demand risks. This provides a new perspective for future research, encouraging scholars to more comprehensively explore the complex relationship between policy and market environments. From a practical standpoint, this study offers important guidance for policymakers and industry practitioners. The results show that strict environmental policies help green building material enterprises maintain sales, prices, and profitability amid market demand risks, offering concrete policy foundations for the industry's sustainable development. Policymakers should consider strengthening the response mechanisms to market demand risks when designing environmental policies, enabling enterprises to invest in and innovate green technologies even under conditions of uncertainty.

### 4.3 Limitations and future research directions

This study offers valuable insights into the interaction between market demand risk and environmental policies in the green building materials sector, particularly in cement; However, it has some limitations. First, the analysis mainly focuses on data from the homogenous green cement industry in China, which may limit the results’ applicability to other sectors. Future research could expand to heterogeneous market, other countries, and other green building materials, such as green wall materials and solar heating systems, to compare the impact of environmental policies across industries and examine how industry characteristics influence the relationship between green degree, price, sales, and profits.

Second, the study does not fully consider external factors, such as changes in the economic environment and consumer behavior. These factors, alongside policy changes, may significantly affect product demand. For instance, consumer preferences, fluctuations in green product prices, and macroeconomic policy adjustments can interact with environmental policies to influence market demand and company strategies. Future research could explore the long-term impact of these external factors on the green building materials sector.

### 4.4 Policy recommendations

This strict environmental policy should be promoted and implemented, and the scope of the pilot program for including low-carbon indicators in government procurement building material standards should be extended. The first batch of pilot cities incorporating low-carbon indicators into Chinese government procurement building material standards consisted of six cities; by 2022, the number of pilot cities expanded to 48, with the goal of having no fewer than 100 pilot cities by 2026. The expansion of the pilot program should be accelerated as both theoretical research findings and pilot cases demonstrate that this strict environmental policy can effectively improve the industry's overall green level and encourage enterprises nationwide to invest in green technologies.

The low-carbon indicator standards for government procurement of building materials should be gradually increased in phases. As overly strict policies may burden enterprises in the short term, the government is recommended to adopt a phased implementation approach when formulating environmental policies. This would gradually raise environmental standards, giving enterprises time to adapt and transform while avoiding sudden cost pressures. For example, during the initial phase, relatively lenient green standards can be set, and as market demand for green products increases, the environmental requirements can be gradually raised.

While enforcing strict environmental policies, local governments should provide targeted research and development subsidies for green upgrades to enterprises affected by market demand shifts. Compared to large cement clinker producers such as Conch Cement, local clinker manufacturers are smaller in scale and have weaker capabilities to cope with the current tightening of market demand, which makes it difficult for them to independently bear the high costs of green upgrades. To maintain a coexistence of various types of enterprises and avoid a situation where small and medium-sized cement clinker companies are driven out of the market owing to the dual pressures of market demand risk and environmental policies, local governments should provide targeted R&D subsidies for green upgrades to small and medium-sized cement clinker manufacturers facing actual difficulties, helping them overcome survival challenges.

## 5 Conclusion

This paper focused on the sustainability challenges faced by the green building material industry and enterprises under the dual pressures of environmental policies and market demand risks. We developed a multi-stage game model between green building material producers and intermediate goods suppliers to analyze the sensitivity of product greenness, prices, sales volumes, and the profits of both parties to changes in market demand risks under strict and relaxed environmental policies. We found that strict environmental policies, compared to lenient ones, better support the sustainable development of the green building material industry. They encourage green research and production, ensuring that products maintain high greenness even under high market demand risks. Moreover, strict policies help businesses sustain sales and prices of green materials, thereby securing profits, especially when competing products have lower greenness.

This research fills a gap in understanding the interactive effects of environmental policies and market demand risks, providing a new perspective on how policies influence the sustainable development of green building material enterprises. The findings offer important recommendations for management practice, urging policymakers to consider market demand risks when designing environmental policies to better support the long-term development of green building material enterprises. However, this study has limitations; for example, it is primarily based on data from the cement industry, which may affect the generalizability of the results. Future research could test these findings in different contexts to enhance their reliability.

## Data and code availability statement

Data included in the article/supplementary material is referenced in the article:

## Declaration of generative AI-assisted technologies in the writing process

During the preparation of this work the author(s) used ChatGPT 4.0 to improve language and readability. After using this tool, the author reviewed and edited the content as needed and takes full responsibility for the content of the publication.

## Declaration of competing interest

The authors declare that they have no known competing financial interests or personal relationships that could have appeared to influence the work reported in this paper.

## Appendix A Derivation Process

(1)Derivatives of ${p_{f1}}^{*},{p_{m}}^{*},{g_{1}}^{*}{{,U}_{P1}}^{*}\text{,}$ ${U_{S1}}^{*}$ with respect to α

$$\frac{{{dp}_{f1}}^{*}}{d\alpha }=\frac{3}{2}\frac{{{dp}_{m1}}^{*}}{d\alpha }=\frac{3\mu }{\beta }\frac{{{dg}_{1}}^{*}}{d\alpha }=\frac{-3\mu \left[4\mu \left(Qe^{-\varepsilon r}-\beta g_{2}+\alpha p_{f2}\right)-\left(4\alpha \mu -\beta^{2}\right)p_{f2}\right]}{{\left(4\alpha \mu -\beta^{2}\right)}^{2}}$$

$$\frac{{{dq}_{1}}^{*}}{d\alpha }=\frac{-\mu \left[\beta^{2}\left(Qe^{-\varepsilon r}-\beta g_{2}+\alpha p_{f2}\right)-\alpha \left(4\alpha \mu -\beta^{2}\right)p_{f2}\right]}{{\left(4\alpha \mu -\beta^{2}\right)}^{2}}$$

$$\frac{d{U_{P1}}^{*}}{d\alpha }=\frac{-2\mu^{2}\left(Qe^{-\varepsilon r}-\beta g_{2}+\alpha p_{f2}\right)\left[4\mu \left(Qe^{-\varepsilon r}-\beta g_{2}+\alpha p_{f2}\right)-\left(4\alpha \mu -\beta^{2}\right)p_{f2}\right]}{{\left(4\alpha \mu -\beta^{2}\right)}^{3}}$$

$$\frac{d{U_{S1}}^{*}}{d\alpha }=\frac{-\mu \left(Qe^{-\varepsilon r}-\beta g_{2}+\alpha p_{f2}\right)\left[2\mu \left(Qe^{-\varepsilon r}-\beta g_{2}+\alpha p_{f2}\right)-\left(4\alpha \mu -\beta^{2}\right)p_{f2}\right]}{{\left(4\alpha \mu -\beta^{2}\right)}^{2}}$$

(2)Derivatives of ${p_{f1}}^{*},{p_{m}}^{*},{g_{1}}^{*}{{,U}_{P1}}^{*}\text{,}$ ${U_{S1}}^{*}$ with respect to β

$$\frac{{{dp}_{f1}}^{*}}{d\beta }=\frac{3}{2}\frac{{{dp}_{m1}}^{*}}{d\beta }=\frac{3}{\alpha }\frac{{{dq}_{1}}^{*}}{d\beta }=\frac{3\mu \left[2\beta \left(Qe^{-\varepsilon r}-\beta g_{2}+\alpha p_{f2}\right)-\left(4\alpha \mu -\beta^{2}\right)g_{2}\right]}{{\left(4\alpha \mu -\beta^{2}\right)}^{2}}$$

$$\frac{{{dg}_{1}}^{*}}{d\beta }=\frac{\left(4\alpha \mu +\beta^{2}\right)\left(Qe^{-\varepsilon r}-\beta g_{2}+\alpha p_{f2}\right)-\beta \left(4\alpha \mu -\beta^{2}\right)g_{2}}{{\left(4\alpha \mu -\beta^{2}\right)}^{2}}$$

$$\frac{d{U_{P1}}^{*}}{d\beta }=\frac{2\mu^{2}\left(Qe^{-\varepsilon r}-\beta g_{2}+\alpha p_{f2}\right)\left[2\beta \left(Qe^{-\varepsilon r}-\beta g_{2}+\alpha p_{f2}\right)-\left(4\alpha \mu -\beta^{2}\right)g_{2}\right]}{{\left(4\alpha \mu -\beta^{2}\right)}^{3}}$$

$$\frac{d{U_{S1}}^{*}}{d\beta }=\frac{\mu \left(Qe^{-\varepsilon r}-\beta g_{2}+\alpha p_{f2}\right)\left[\beta \left(Qe^{-\varepsilon r}-\beta g_{2}+\alpha p_{f2}\right)-\left(4\alpha \mu -\beta^{2}\right)g_{2}\right]}{{\left(4\alpha \mu -\beta^{2}\right)}^{2}}$$

(3)Derivatives of ${p_{f1}}^{*},{p_{m}}^{*},{g_{1}}^{*}{{,U}_{P1}}^{*}\text{,}$ ${U_{S1}}^{*}$ with respect to r

$$\frac{{{dp}_{f1}}^{*}}{dr}=\frac{3}{2}\frac{{{dp}_{m1}}^{*}}{dr}=\frac{\mu }{\beta }\frac{{{dg}_{1}}^{*}}{dr}=\frac{3}{\alpha }\frac{{{dq}_{1}}^{*}}{dr}=\frac{-\varepsilon Q∙e^{-\varepsilon r}∙3\mu }{4\alpha \mu -\beta^{2}}$$

$$\frac{d{U_{P1}}^{*}}{dr}=\frac{-2\varepsilon Q∙e^{-\varepsilon r}∙\alpha \mu^{2}\left(Qe^{-\varepsilon r}-\beta g_{2}+\alpha p_{f2}\right)}{\alpha {\left(4\alpha \mu -\beta^{2}\right)}^{2}}$$

$$\frac{d{U_{S1}}^{*}}{dr}=\frac{-\varepsilon Q∙e^{-\varepsilon r}∙\mu \left(Qe^{-\varepsilon r}-\beta g_{2}+\alpha p_{f2}\right)}{4\alpha \mu -\beta^{2}}<0$$

## Footnotes

Appendix A Supplementary data to this article can be found online at https://doi.org/10.1016/j.heliyon.2025.e41942.
