# Supplementary material for: Navigating dual pressures: The impact of environmental policies and market demand risks on the sustainable development of green building materials - A case study of the green cement industry
Source: Heliyon. 2025 Jan 16;11(2):e41942. doi: 10.1016/j.heliyon.2025.e41942 (PMC11791233; doi:10.1016/j.heliyon.2025.e41942)
Supplement: Multimedia component 1 [file mmc1.docx]

## Appendix B: Simulation Code

clear

clc

popSize = 300;

maxGenerations = 100;

crossoverRate = 0.7;

baseMutationRate = 0.01;

gMutationRate = 0.01;

numSimulations = 100;

eliteCount = 2;

a = 1; u = 1; e = 2.71828; y=1; Q = 1; Dp = 0; Ds = 0;

%b = 5.5; pj2 = 0.65; g2 = 0.45; %1

b = 5.5; pj2 = 0.45; g2 = 0.35; %5

r1_values = linspace(0, 5, 50);

g1_values = zeros(numSimulations, length(r1_values));

q1_values = zeros(numSimulations, length(r1_values));

pj1_values = zeros(numSimulations, length(r1_values));

pm1_values = zeros(numSimulations, length(r1_values));

U_P1_values = zeros(numSimulations, length(r1_values));

for r = 1:length(r1_values)

r1 = r1_values(r);

for sim = 1:numSimulations

population = zeros(popSize, 3);

population(:, 1) = 0.3 + 0.4 * rand(popSize, 1); % pj1: 300-400

%population(:, 2) = 300 + 100 * rand(popSize, 1); % pj2: 300-400

population(:, 2) = 0.2 + 0.3 * rand(popSize, 1); % pm1: 200-300

%population(:, 4) = 200 + 100 * rand(popSize, 1); % pm2: 200-300

population(:, 3) = 0.3 + 0.4 * rand(popSize, 1); % g1: 0-1

%population(:, 6) = 300 + 100 * rand(popSize, 1); % g2: 0-1

bestFitness = zeros(maxGenerations, 1);

meanFitness = zeros(maxGenerations, 1);

for generation = 1:maxGenerations

fitness = arrayfun(@(i) objectiveFunction(population(i, :), r1, b, pj2, g2), 1:popSize);

[~, sortedIndices] = sort(fitness, 'descend');

eliteIndividuals = population(sortedIndices(1:eliteCount), :);

parentIndices = rouletteWheelSelection(fitness, popSize - eliteCount);

parents = population(parentIndices, :);

children = crossover(parents, crossoverRate);

adaptiveMutationRate = baseMutationRate * (1 - generation / maxGenerations);

children = mutate(children, adaptiveMutationRate, gMutationRate);

population = [eliteIndividuals; children];

end

[~, bestIndex] = max(fitness);

optimalSolution = population(bestIndex, :);

pj1 = optimalSolution(1);

%pj2 = optimalSolution(2);

pm1 = optimalSolution(2);

%pm2 = optimalSolution(4);

g1 = optimalSolution(3);

%g2 = optimalSolution(6);

q1 = Q * e^(-y*r1) - a * (pj1 - pj2) + b * (g1 - g2);

U_P1 = (pj1 - pm1 - Dp) * q1;

U_S1 = (pm1 - Ds) * q1 - (u * g1^2) / 2;

g1_values(sim, r) = g1;

q1_values(sim, r) = q1;

pj1_values(sim, r) = pj1;

pm1_values(sim, r) = pm1;

U_P1_values(sim, r) = U_P1;

U_S1_values(sim, r) = U_S1;

end

end

mean_g1 = mean(g1_values, 1);

mean_q1 = mean(q1_values, 1);

mean_pj1 = mean(pj1_values, 1);

mean_pm1 = mean(pm1_values, 1);

mean_U_P1 = mean(U_P1_values, 1);

mean_U_S1 = mean(U_S1_values, 1);

Ga1 = [mean_g1; mean_q1; mean_pj1; mean_pm1; mean_U_P1; mean_U_S1];

save('Ga1.mat', 'Ga1');

function selectedIndices = rouletteWheelSelection(fitness, numToSelect)

cumulativeFitness = cumsum(fitness) / sum(fitness);

selectedIndices = zeros(numToSelect, 1);

for i = 1:numToSelect

r = rand;

selectedIndices(i) = find(cumulativeFitness >= r, 1, 'first');

end

end

function offspring = crossover(parents, crossoverRate)

[numParents, numVars] = size(parents);

offspring = zeros(numParents, numVars);

for i = 1:2:numParents-1

if rand < crossoverRate
